# Do menstrual symptoms affect motor imagery skills in young women?

**DOI:** 10.1007/s00404-025-07936-5

**Published:** 2025-01-31

**Authors:** Özgü İnal Özün, Merve Öztürk, Esra Üzelpasacı

**Affiliations:** 1 Faculty of Gülhane Physiotherapy and Rehabilitation, Department of Neurological Physiotherapy and Rehabilitation, University of Health Sciences, Ankara, Turkey; 2 Ankara Etlik City Hospital, Physical Therapy and Rehabilitation Hospital, Ankara, Turkey; 3 Faculty of Gülhane Physiotherapy and Rehabilitation, Department of Cardiopulmonary Physiotherapy and Rehabilitation, University of Health Sciences, Ankara, Turkey

**Keywords:** Menstrual symptoms, Motor imagery, Pain, Somatic complaints

## Abstract

**Purpose:**

To examine the relationship between menstrual symptoms and motor imagery skills in young women.

**Methods:**

A total of 117 women between the ages of 18–40 were included in the study. Visual Analog Scale (VAS) for the menstrual pain intensity, the Menstrual Symptom Questionnaire (MSQ) for the menstrual-related symptoms, and the Kinesthetic and Visual Imagery Questionnaire-20 (KVIQ-20) for the motor imagery were used. All measurements were conducted on the first or second day of the menstruation, depending on when the subject declared subjectively experiencing most symptoms.

**Results:**

The relationship between menstruation symptoms and visual imagery (VI) and kinesthetic imagery (KI) skills was examined. A weak negative correlation was found between MSQ_negative effects/somatic complaints and KVIQ-20_KI (*p* = .040, *r* = −.199). The relationship between menstrual pain intensity and KVIQ-20 items was examined. The strongest relationship was found between VAS and KVIQ-20_knee_VI and KVIQ-20_hip_VI (*p* = 003, *r* = −.288; *p* = 005, *r* = −.270; *p* = 004, respectively). A weak negative correlation was also found between VAS and KVIQ-20_VI_total and KVIQ-20_KI_total (*p* = 0.004, *r* = −.275; *p* = 0.19, *r* = −.227, respectively).

**Discussion:**

This is the first study to examine menstrual symptoms in women in detail and reveal their relationship with motor imagery skills. Menstrual symptoms, especially negative effects/somatic complaints seen during menstruation, negatively affect kinesthetic imagery ability. In addition; chronic menstrual pain has a negative effect on both kinesthetic and visual imagery abilities. Considering the impairments in motor imagery skills due to negative effects/somatic complaints during menstruation and chronic menstrual pain, adding motor imagery training to treatment programs aimed at improving women’s health may have positive effects.

## What does this study add to the clinical work

**Table:** 

This is the first study to examine the relationship between menstrual symptoms and motor imagery skills in women. The study findings provide evidence that increased negative affect/somatic complaints from menstrual symptoms reduce kinesthetic imagery ability. Furthermore, chronic menstrual pain has a negative effect on both kinesthetic and visual imagery abilities. Adding motor imagery training to treatment programs designed to improve women's health may have positive effects.	

## Introduction

Menstrual cycle is a physiological process that occurs approximately every 28 days in women of reproductive age due to hormonal fluctuations. Studies have shown that women may experience physical and mental symptoms due to hormone fluctuations that occur during the menstrual cycle [1]. The most common of these symptoms are perimenstrual mood disorders (frequently irritability, tension, anger, etc.) and somatic symptoms (e.g., breast tenderness, heavy menstrual bleeding, dysmenorrhea, abdominal bloating, headache and limb edema) [2]. All these menstrual symptoms negatively affect women’s daily lives, academic and sports performance. In addition, hormonal changes seen during menstruation may affect cognitive and psychomotor performance changes and may also alter executive functions [3, 4].

Motor imagery (MI) is one of the practices that have an effect on motor performance. MI is the act of imagining the movement without actually performing it. MI is a movement representation technique that causes brain regions involved in the planning, generation, adjustment, and automation of voluntary movement to be activated in much the same way as when the action is actually performed [5]. For a variety of tasks (such as motor sequencing, motor timing, strenght, flexibility of the motor system) the benefits of motor imagery practice have been demonstrated [6]. Researches on the use of MI in women’s health have begun to increase in the last few years [7, 8]. There are two main types of MI; kinesthetic motor imagery (KMI) and visual motor imagery (VMI). KMI is defined as the ability to imagine performing a movement using an impression of the muscle contraction and sensation during an actual movement. VMI is the ability to imagine seeing oneself performing the movement [9]. MI is an important tool used in rehabilitation to improve motor performance skills [10], and therefore, it is important to understand the mechanisms associated with MI.

Studies in the literature mostly focus on changes in motor skills across the menstrual cycle and menstrual pain symptom and their negative effects on cognitive abilities. Ronca et al. [11] have suggested that visuospatial and anticipatory processes may fluctuate throughout the menstrual cycle in the general population, with better performance during the menstrual phase and poorer performance during the luteal phase. It has also been emphasized that this cognitive changes specific to menstrual phase may increase the risk of injury in women, in addition to biomechanical factors. A recent study [12] indicated that fine motor skills vary across the phases of the menstrual cycle and that this may be modulated by sensorimotor integration and menstruation-related symptoms. However, there is no study that examines menstrual symptoms in detail and reveals their relationship with both motor and visual cognitive abilities.

Measuring motor imagery ability and determining the factors affecting motor imagery ability before starting training in the rehabilitation process of women is a very important factor in terms of the trainings to be given [13]. This study was planned to understand the relationship between menstrual symptoms and motor imagery skills in young women. It is thought that the findings of the study will be guiding in terms of women’s health rehabilitation studies.

## Materials and methods

The study was approved by the Ethics Committee of the Çankırı Karatekin University Health Sciences (decision date: 03–04-2024; meeting number: 12). This study was a cross-sectional study and participants were reached by snowball sampling method [14]. Literate women between the ages of 18–40, with a menstrual cycle, without any diagnosed neurologic, orthopaedic and gynecologic disease and have no history of taking oral contraceptives for 3 months or longer were included in the study. Women with a history of pregnancy and childbirth, urogynecologic disease, pelvic surgery, diagnosed chronic pain and psychiatric disease were excluded from the study. The study was carried out in accordance with the Helsinki Declaration and written voluntary consent form was taken from the participants included in the study. The socio-demographic information form prepared by the researchers, the Visual Analog Scale for the assessment of menstrual pain intensity, the Menstrual Symptom Questionnaire (MSQ) for the assessment of menstrual-related symptoms, and the Kinesthetic and Visual Imagery Questionnaire-20 (KVIQ-20) for the assessment of motor imagery were used. All measurements were conducted during a single menstrual cycle, specifically on the first or second day of the menstruation, depending on when the subject declared subjectively experiencing most symptoms.

***The visual analog scale (VAS);*** a 100-mm visual analog scale (VAS) ranging from "no pain at all" to "the worst pain I have ever felt" was used to assess the degree of each women’s dysmenorrheic pain [15]. The distance between the marked point and the starting point was measured in cm. An increase in the score means an increase in the severity of pain [16].

***Menstrual symptom questionnaire;*** was developed by Chesney and Tasto in 1975 to assess menstrual pain and symptoms [17]. Its Turkish validity and reliability were conducted by Güvenç et al. in 2014 [18]. MSQ is a five-point Likert-type scale consisting of 22 items. Participants are asked to give a number between 1 (never) and 5 (always) to the symptoms they experience related to menstruation. The scale has three sub-dimensions: “negative effects/somatic complaints” (items 1–13), “menstrual pain symptoms” (items 14–19) and “coping methods” (items 20–22). An increase in the mean score indicates an increase in the severity of menstrual symptoms. Cronbach’s Alpha value of the scale was found to be 0.89.

***Kinesthetic and visual ımagery questionnaire-20 (KVIQ-20);*** consists of a total of 10 movements, five of which are visual and five of which are kinesthetic imagery skills, and was developed to determine the extent to which individuals visualize and feel the imagined movements [19, 20]. A high score is considered an indicator of a good level of imagery. The Cronbach’s Alpha value of the scale is high (test–retest: 0.98–0.98). The questionnaire is not a self-report scale and is administered with an assessor. The assessment was performed under the guidance of a physiotherapist. All movements were assessed in a sitting position. The assessor first performed the movement on himself and then asked the participant to perform the same movement only once. The participant then visualized the movement and rated the visual clarity or intensity of the sensations using a five-point ordinal scale.

### Statistical analysis

Data were analyzed using SPSS (Statistical Package for Social Sciences) for the Windows 25.0 program. Descriptive statistics such as frequency and percentage values were used regarding the obtained information. The study variables’ Kurtosis and Skewness values were analyzed to see if they displayed a normal distribution. The Pearson correlation analysis was used to determine the relationship between three scale scores. The following cutoff points were used for the interpretation of correlation coefficients: 0.00–0.19 very weak, 0.20–0.39 weak, 0.40–0.69 moderate, 0.70–0.89 high and 0.90–1.00 very high. Statistical significance was accepted as *p* < 0.05 [21].

## Results

A total of 117 participants were screened for eligibility. 11 participants did not meet the inclusion criteria (chronic diseases [*n* = 5], having another accompanying chronic pain [*n* = 3], and unwillingness to participate in study [*n* = 3]). Consequently, the study was condcted with 106 female subjects with a mean age of 25.5 ± 4.9 years. Descriptive characteristics of the participants are given in Table 1.

**Table 1:** Descriptive data (*N* = 106)

		Min–max	Mean ± SD
Age	Year	18–40	25.5 ± 4.9
Age of first menstruation	Year	11–20	13.3 ± 1.4
		*N* = 106	%
Education	Primary school	1	0.9
	Secondary school	2	1.9
	High school	5	4.7
	Undergraduate	95	89.6
	Postgraduate	3	2.8
Smoking	No	91	85.8
	1–10 years	9	8.5
	10–20 years	6	5.7
Alcohol consumption	No	87	82.1
	Sometimes	18	17.0
	Frequently	1	0.9
Occupation	Student	40	37.7
	Public	58	54.7
	Private industry	8	7.5
Intrauterine device use	Yes	1	0.9
	No	105	99.1
Regular exercise	Yes	27	25.5
	No	79	74.5
Menstrual cycle	< 21 day	7	6.6
	21–35 day	93	87.7
	> 35 day	6	5.7
Menstruation duration	< 2 day	2	1.9
	2–7 day	90	84.9
	> 7 day	14	13.2
Symptoms	Headache	39	36.8
	Vomiting	4	3.8
	Fatigue	42	39.6
	Diarrhea	12	11.3
Pain medication use time	Before menstruation	5	4.7
	Menstruation day 1	60	56.6
	Other	41	38.7
Other methods	Heat applications (e.g. hotpack)	78	73
	Herbal product and/or vitamin	16	15.1
	No	12	11.3

In the study, the relationship between menstruation symptoms and visual and kinesthetic imagery skills was examined. No relationship was found between MSQ sub-dimensions and KVIQ-20_VI skills. A weak negative correlation was found between MSQ_1 and KVIQ-20_KI (*p* = 0.040, *r* = −0.199) and between MSQ_1 and KVIQ-20_total (*p* = 0.048, *r* = −0.193) (Table 2).

**Table 2:** Correlation between the menstrual symptom scale and the kinesthetic and visual imagery imagery questionnaire-20

	MSQ_1		MSQ_2		MSQ_3	
	*p*	*r*	*p*	*r*	*p*	*r*
KVIQ-20_VI	.060	−.183	.079	−.172	.201	−-.125
KVIQ-20_KI	**.040**	−.199	.197	−.126	.133	−.147
KVIQ_Total	**.048**	−.193	.124	−.150	.152	−.140

Those written in bold indicate p < 0.05

*KVIQ-20*: Kinesthetic and Visual Imagery Questionnaire-20, *VI*: Visual Imagery, *KI*: Kinesthetic Imagery, *MSQ*: Menstrual Symptom Questionnaire, MSQ**_** 1: negative effects/somatic complaints, MSQ_2: menstrual pain symptoms, MSQ_3: coping methods, Pearson Correlation Analysis; *p* < 0.05

In the study, the relationship between menstrual pain intensity (the intensity of pain experienced during menstruation in the last 6 months) and kinesthetic and visual imagery questionnaire items was examined. The strongest relationship was found between VAS and KVIQ-20_knee_VI and KVIQ-20_hip_VI (*p* = 003, *r* = −0.288; *p* = 005, *r* = −0.270; *p* = 004, respectively). A weak negative correlation was also found between VAS and KVIQ-20_VI_total and KVIQ-20_KI_total (*p* = 0.004, *r* = −0.275; *p* = 0.19, *r* = −0.227, respectively) (Table 3).

**Table 3:** Correlation between menstrual pain intensity in the last 6 months and kinesthetic and visual imagery questionnaire-20 items

	VAS	
	*p*	*r*
KVIQ-20_neck_VI	**.039**	**−.201**
KVIQ-20_ neck_KI	**.016**	**−.233**
KVIQ-20_shoulder_VI	**.007**	**−.262**
KVIQ-20_ shoulder_KI	**.042**	**−.198**
KVIQ-20_arm_VI	**.030**	**−.211**
KVIQ-20_ arm_KI	**.031**	**−.210**
KVIQ-20_elbow_VI	**.006**	**−.258**
KVIQ-20_ elbow_KI	**.006**	**−.263**
KVIQ-20_thumb_VI	**.016**	**−.233**
KVIQ-20_ thumb_KI	.100	**−**.161
KVIQ-20_trunk_VI	**.032**	**−.209**
KVIQ-20_ trunk_KI	.106	**−**.158
KVIQ-20_knee_VI	**.003**	**−.288**
KVIQ-20_ knee_KI	**.018**	**−.230**
KVIQ-20_hip_VI	**.005**	**−.270**
KVIQ-20_ hip_KI	**.015**	**−.263**
KVIQ-20_ foot tapping_VI	**.002**	**−.234**
KVIQ-20_ foot tapping_KI	**.042**	**−.198**
KVIQ-20_foot external rotation_VI	**.029**	**−.148**
KVIQ-20_ foot external rotation_KI	.181	**−**.131
KVIQ-20_VI_total	**.004**	**−.275**
KVIQ-20_KI_total	**.019**	**−.227**
KVIQ-20_total	**.007**	**−.263**

Those written in bold indicate p < 0.05

*VAS* Visual Analog Scale, *KVIQ-20* Kinesthetic and Visual Imagery Questionnaire-20, *VI* Visual Imagery, *KI* Kinesthetic Imagery, Pearson Correlation Analysis; *p* < 0.05

## Discussion

This study examined the relationship between menstrual symptoms and motor imagery skills in young women. In this study, it has been determined that the negative relationship between menstrual symptoms with negative effects/somatic and KI. In the study, it was also determined that the average of menstrual pain intensity in the last 6 months was negatively associated with kinesthetic and VI skills. This is the first study to examine menstrual symptoms in women in detail and reveal their relationship with motor imagery skills.

It has been reported that 50–90% of women experience psychological or physical symptoms during menstruation and these symptoms affect cognitive and motor performance [22]. The present study has shown that as negative effects and somatic symptoms decreased, the young women’s KI skill increased. Negative effects such as irritability, tension, anger and somatic findings such as breast tenderness, abdominal distension, headache and extremity edema are commonly seen in women during menstrual period [2]. There is no study in the literature on the relationship between menstruation symptoms and MI ability, but there are some studies on the relationship between negative effects and somatic symptoms and MI in different situations. In particular, there is evidence that musculoskeletal, neuropathic and visceral pain all contribute to the misperception of body parts (e.g., the feeling that some body parts are in a different position than the actual position) [23]. Examination of motor imagery as a cognitive process in the context of sport reveals that it has a significant impact on skill development, performance optimization, and the overall psychological well-being of athletes [24]. In a study examining the relationship between imagery and competitive anxiety in ballet auditions [25], KI ability contributed to variation in cognitive anxiety and confidence; dancers with higher ability reported higher confidence and lower anxiety. In a study examining the relationship between visual and kinesthetic imagery skills and performance, it was shown that VI ability predicted both cognitive and somatic issues, while KI ability predicted state-sport confidence and was associated with better performance [25, 26]. In a study involving individuals who had suffered spinal cord injuries [27], psychological factors were found to be associated with mental imagery.

In the present study, a negative relationship was found between negative effects/somatic complaints and KI, but no relationship was found between VI. Kinesthetic awareness involves a “whole body scan” in which the person scans their body for tension, imagines movements, and then imagines performing the action [28]. Many of the somatosensory and psychological symptoms of the menstrual cycle are significantly associated with body dissatisfaction. These include water retention and its complications (e.g., weight gain, bloating, and breast tenderness), pain, negative mood, increased appetite, and changes in visual and olfactory thresholds [29]. Impaired awareness of one’s own body shape and organs may result from lack of proprioception and motor imagery (lateralization) [30]. Because motor imagery is a process that activates the working body schema [31]. Therefore, in the present study, a relationship was found between negative effects/somatic complaints and KI skill, whereas no relationship was found between VI skill. Another reason is that different brain networks mediate kinesthetic and visual imagery [32]. A study using magnetoencephalography (MEG) to investigate the difference in brain activation between VI and KI [33] found that activation of the frontal cortex was significantly suppressed during KI compared to visual imagery. This suggests that more cognition and information processing may be involved during KI [34]. In subjects with good to excellent motor imagery ability, both types of motor imagery were found to activate the primary motor cortex, but kinesthetic motor imagery shared more neural pathways with motor execution [32]. It is thought that this may be a factor in the association of KI skill with menstrual symptoms. In addition to all these inferences, the fact that there is evidence that somatic therapy practices in which the client uses a combination of interoceptive and kinesthetic imagery by focusing on the client’s inner attention can help in the treatment of negative symptoms [35] supports the relationship between negative effects/somatic complaints and KI skill in the current study.

In this study, the relationship between menstrual pain intensity and kinesthetic and visual imagery questionnaire items was examined. As pain intensity increased, visual and kinesthetic imagery of almost all anatomical structures decreased. Pain is a complex, disturbing event that affects both cognitive and motivational–emotional domains and thus the behavior of those who experience it [36]. Besides physiological evidence of impaired somatotopic representation in chronic pain, there is also behavioral evidence of impaired spatial representation: impaired processing of stimuli transmitted to healthy body parts held in the affected area, abnormal perceived size of the painful body part, and poor voluntary movement and motor imagery performance [37, 38]. A study in young adults with chronic neck pain [39] found that motor imagery ability was reduced and that this impairment was associated with the severity of disability. In the same study, one unit of the sensory effect of pain decreased kinesthetic imagery by 0.09 units. La Touche et al. (2019) [40] found that patients with chronic pain had more difficulty in producing both kinesthetic and visual mental motor images compared to symptom-free participants. This finding suggests that patients with chronic pain have less ability to produce mental motor images. There is evidence that people with chronic pain tend to perceive their painful areas/body parts as enlarged or shrunk [38, 41]. In a study by Bowering et al. in patients with low back pain [42], it was found that trunk motor imagery performance decreased during a current episode. This was associated with impaired cortical proprioceptive representation of the trunk. Similar results were obtained from Bray and Moseley’s study (2011) [43] and it was determined that back pain caused motor imagery deficits.

In the present study, the strongest relationship was found between the average severity of menstrual pain in the last 6 months and knee and hip MI skills. Cortical proprioceptive maps are maintained by tactile, proprioceptive and visual inputs [44]. There is evidence in the literature that visual, tactile and auditory stimuli feed into the cortical proprioceptive representation, but unlike the hands or face, these sensory inputs do not normally occur back. In addition, it has been shown that the back region may be subject to greater loss of accuracy in motor imagery tasks than the hands, because there are no other sensory modalities to reinforce the representation. Especially in daily activities, the fact that the hands and feet are frequently visualized and have almost continuous tactile input during use has been associated with less motor imagery task losses compared to other body parts [45, 46]. In the present study, the fact that the relationship between pain intensity and MI was most impaired in the knee and hip may be related to both the closeness of the anatomical relationship of pelvic structures with the knee and hip, and the fact that the knee and hip are exposed to less sensory input in daily life, as in the back region. It has been noted in the literature that in chronic syndromes (i.e., complex regional pain syndrome), pain affects the mental imagery of movement, but only when actions are related to painful body parts [42, 47]. In addition, it is known that different body parts are located in different areas and with different representations in the primary somatosensory cortex [48], and therefore, it seems reasonable that different body parts are represented by different patterns in cortical proprioceptive maps [42]. The present study is the first to examine the relationship between pain intensity and MI skill regionally. More studies examining the relationship between pain intensity and MI regionally are needed to further explain the findings related to the results.

In the present study, no relationship was found between MSQ_menstrual pain symptoms and visual and kinesthetic imagery skills, whereas a relationship was found between VAS scores questioning menstrual pain intensity and both visual and kinesthetic imagery skills. MSQ_menstrual pain symptoms sub-dimension items assess findings related to pain during menstruation, while VAS pain intensity assesses the average pain intensity of the individual during menstruation in the last 6 months. Considering the complex nature of pain, it is thought that different parameters related to pain (e.g., severity, duration, type, and symptoms) may affect MI skill differently, so these parameters should be evaluated in detail in studies.

MI techniques have been proven to reduce pain associated with phantom limb pain, complex regional pain syndrome and other types of pain with unknown pathology [49]. In recent years, methods involving body representations such as MI have been widely used in women’s health studies and positive effects of these methods have been reported [50]. In this context, examining the relationship between menstrual symptoms and MI skills is thought to be guiding in terms of developing strategies for symptom management.

### Strenghts and limitation

Healthy individuals without any disease diagnosis were included in the study. We relied entirely on self-reporting to withhold health information for inclusion and exclusion criteria. Studies with healthy participants show that even in the absence of brain lesions, information from the body may be misleading and create bodily illusions [51]. Excluding the participants from gynecological problems based solely on their history may have caused an undiagnosed disease to be overlooked. This situation is a limitation of the study. In terms of determinants such as age and gender [52] that make a difference in the cortical system, our study consists of women between the ages of 18–40. This is considered as a strong aspect in terms of homogenization of the study results. However, the differences created by different ages in the cortical system may also affect the results of future studies. Imagery is multidimensional and the main areas are vividness, controllability, accuracy, duration and ease [27]. In the present study, vividness and accuracy of imagery were assessed. However, the questionnaires used were limited in scope to assess other aspects of imagery, such as controllability, ease of imagery or emotional aspects. Furthermore, the association of menstrual symptoms with imagery ability was investigated, but the different cognitive resources involved in imagery were not examined separately (e.g., psychological parameters). These are other limitations of the study. Future research should specifically investigate the relationship between MI and the parameters encompassed by the concept of negative effects/somatic complaints (e.g., irritability, tension, and limb edema). In addition, premenstrual symptoms were not included specifically in our assessment as the primary focus of our study was to examine the general effects of menstrual symptoms on motor imagery. While the MSQ includes questions addressing premenstrual symptoms, a more detailed evaluation may reveal additional insights. Future studies should consider a more focused analysis of premenstrual symptoms and their potential influence on motor imagery performance.

## Conclusions

The results of this study demonstrated that menstrual symptoms, especially negative effects/somatic complaints seen during menstruation, negatively affect kinesthetic imagery ability. In addition; chronic menstrual pain has a negative effect on both kinesthetic and visual imagery abilities. Considering the impairments in motor imagery skills due to negative effects/somatic complaints during menstruation and chronic menstrual pain, adding motor imagery training to treatment programs aimed at improving women’s health may have positive effects.

## Data Availability

The data used in this study are available upon request from the corresponding author.

## References

[1] Nuria RP, Rocío C, Alfaro-Magallanes Victor M, Beatriz Rael, Rubio-Arias Jacobo Á, Peinado Ana B, Benito Pedro J (2021) Exercise-ınduced muscle damage during the menstrual cycle: a systematic review and meta-analysis. J Strength Cond Res 35(2):549–561. 10.1519/JSC.0000000000003878 33201156 10.1519/JSC.0000000000003878

[2] Negriff S, Dorn LD, Hillman JB, Huang B (2009) The measurement of menstrual symptoms: factor structure of the menstrual symptom questionnaire in adolescent girls. J Health Psychol 14(7):899–908. 10.1177/1359105309340995 19786516 10.1177/1359105309340995 PMC4301608

[3] Prabhavathi K, KalyaniPraba P, Rajendra BN, Saravanan A (2023) Cognition and psychomotor performance in premenstrual syndrome with negative emotions. Biomed Pharm J 16(4):2061–2067. 10.13005/bpj/2782

[4] Kluska J, Malinowska E, Kowalski J (2024) A pilot longitudinal study of decrease in cognitive functions during the most painful day of the period among women with primary dysmenorrhea. Arch Gynecol Obstet. 10.1007/s00404-024-07617-9 38963585 10.1007/s00404-024-07617-9 PMC11890368

[5] Taube W, Mouthon M, Leukel C, Hoogewoud HM, Annoni JM, Keller M (2015) Brain activity during observation and motor imagery of different balance tasks: an fMRI study. Cortex 64:102–114. 10.1016/j.cortex.2014.09.022 25461711 10.1016/j.cortex.2014.09.022

[6] Ladda AM, Lebon F, Lotze M (2021) Using motor imagery practice for improving motor performance–a review. Brain Cogn 150:105705. 10.1016/j.bandc.2021.105705 33652364 10.1016/j.bandc.2021.105705

[7] Cuenca-Martínez F, Nieves-Gómez A, Millán-Isasi N, Fuentes-Aparicio L, Sempere-Rubio N (2025) Effects of motor imagery and action observation on pelvic floor and related structures in healthy women: a randomized controlled trial. Hum Mov Sci 99:103313. 10.1016/j.humov.2024.103313 39626586 10.1016/j.humov.2024.103313

[8] Yakıt Yeşilyurt S, Birinci Olgun T, Ayaz Taş S, Tosun G, Özer M, Özengin N (2024) Safety and efficacy of motor imagery-based physical activity in high-risk pregnancy: a randomized controlled study. Int J Gynaecol Obstet 167(3):1222–1230. 10.1002/ijgo.15799 39031032 10.1002/ijgo.15799

[9] Yang YJ, Jeon EJ, Kim JS, Chung CK (2021) Characterization of kinesthetic motor imagery compared with visual motor imageries. Sci Rep 11(1):3751. 10.1038/s41598-021-82241-0 33580093 10.1038/s41598-021-82241-0 PMC7881019

[10] Yoxon E, Welsh TN (2019) Rapid motor cortical plasticity can be induced by motor imagery training. Neuropsychologia 134:107206. 10.1016/j.neuropsychologia.2019.107206 31563576 10.1016/j.neuropsychologia.2019.107206

[11] Ronca F, Blodgett JM, Bruinvels G, Lowery M, Raviraj M, Sandhar G, Symeonides N, Jones C, Loosemore M, Burgess PW (2024) Attentional, anticipatory and spatial cognition fluctuate throughout the menstrual cycle: Potential implications for female sport. Neuropsychologia. 10.1016/j.neuropsychologia.2024.108909 38762068 10.1016/j.neuropsychologia.2024.108909

[12] Ikarashi K, Sato D, Edama M, Fujimoto T, Ochi G, Yamashiro K (2024) Fluctuation of fine motor skills throughout the menstrual cycle in women. Sci Rep 14:15079. 10.1038/s41598-024-65823-6 38956128 10.1038/s41598-024-65823-6 PMC11219923

[13] Jackson PL, Lafleur MF, Malouin F, Richards C, Doyon J (2001) Potential role of mental practice using motor imagery in neurologic rehabilitation. Arch Phys Med Rehabil 82(8):1133–1141. 10.1053/apmr.2001.24286 11494195 10.1053/apmr.2001.24286

[14] Handcock MS, Gile KJ (2011) Comment: on the concept of snowball sampling. Sociol Methodol 41(1):367–371. 10.1111/j.1467-9531.2011.01243.x 35095124 10.1111/j.1467-9531.2011.01243.x PMC8797839

[15] Iacovides S, Baker FC, Avidon I (2014) The 24-h progression of menstrual pain in women with primary dysmenorrhea when given diclofenac potassium: a randomized, double-blinded, placebo-controlled crossover study. Arch Gynecol Obstet 289:993–1002. 10.1007/s00404-013-3073-8 24190696 10.1007/s00404-013-3073-8

[16] Revil SI, Robinson JO, Rosen M (1976) The reialility of a linear analogue for evaluation pain. Anaesthesia 31(9):1191–1198. 10.1111/j.1365-2044.1976.tb11971.x 1015603 10.1111/j.1365-2044.1976.tb11971.x

[17] Chesney MA, Tasto DL (1975) The development of the menstrual symptom questionnaire. Behav Res Ther 13(4):237–244. 10.1016/0005-7967(75)90028-5 1238078 10.1016/0005-7967(75)90028-5

[18] Güvenç G, Seven M, Akyüz A (2014) Adaptation of the menstruation symptom scale into Turkish. TAF Prev Med Bull 13(5):367–374. 10.1097/10.5455/pmb1-1378840527

[19] Malouin F, Richards CL, Jackson PL, Jackson PL, Lafleur MF, Durand A, Doyon J (2007) The kinesthetic and visual imagery questionnaire (KVIQ) for assessing motor imagery in persons with physical disabilities: a reliability and construct validity study. J Neurol Phys Ther 31(1):20–29. 10.1097/01.NPT.0000260567.24122.64 17419886 10.1097/01.npt.0000260567.24122.64

[20] Dilek B, Ayhan Ç, Yakut Y (2019) Validity and reliability of the Turkish version of the kinesthetic and visual imagery questionnaire- 20. JETR 6(3):201–210

[21] Alpar R (2016). Applied statistics and validity-reliability with examples from sports, health and educational sciences. Ankara, Turkey: Detay.

[22] Hoyer J, Burmann I, Kieseler ML, Vollrath F, Hellrung L, Arelin K, Roggenhofer E, Villringer A, Sacher J (2013) Menstrual cycle phase modulates emotional conflict processing in women with and without premenstrual syndrome (PMS)–a pilot study. PLoS ONE 8(4):e59780. 10.1371/journal.pone.0059780 23637739 10.1371/journal.pone.0059780 PMC3634788

[23] Scandola M, Pietroni G, Landuzzi G, Polati E, Schweiger V, Moro V (2022) Bodily illusions and motor imagery in fibromyalgia. Front Hum Neurosci 15:798912. 10.3389/fnhum.2021.798912 35126075 10.3389/fnhum.2021.798912 PMC8811121

[24] Tuli K (2023) Unlocking athletic excellence: the cognitive power of motor imagery in sports. IJIAP 1(5):19–31

[25] Monsma EV, Overby LY (2004) The relationship between imagery and competitive anxiety in ballet auditions. J Dance Med Sci 8(1):11–18

[26] Callow N, Waters A (2005) The effect of kinesthetic imagery on the sport confidence of flat-race horse jockeys. Psychol Sport Exerc 6(4):443–459. 10.1016/j.psychsport.2004.08.001

[27] Kaur J, Ghosh S, Singh P, Dwivedi AK, Sahani AK, Sinha JK (2022) Cervical spinal lesion, completeness of injury, stress, and depression reduce the efficiency of mental imagery in people with spinal cord injury. Am J Phys Med Rehabil 101(6):513–519. 10.1097/PHM.0000000000001955 35034059 10.1097/PHM.0000000000001955

[28] Sisterhen LA (2005). The use of imagery, mental practice, and relaxation techniques for musical performance enhancement. The University of Oklahoma. https://www.proquest.com/docview/276093741?pq-origsite=gscholar&fromopenview=true&sourcetype=Dissertations%20&%20Theses Accessed 25 Aug 2024

[29] Carr-Nangle RE, Johnson WG, Bergeron KC, Nangle DW (1994) Body image changes over the menstrual cycle in normal women. Int J Eat Disord 16(3):267–273. 10.1002/1098-108X(199411)16:3%3c267::AID-EAT2260160307%3e3.0.CO;2-Y 7833960 10.1002/1098-108x(199411)16:3<267::aid-eat2260160307>3.0.co;2-y

[30] Lotze M, Moseley GL (2007) Role of distorted body image in pain. Curr Rheumatol Rep 9(6):488–496 18177603 10.1007/s11926-007-0079-x

[31] Ataria Y, Tanaka S, Gallagher S (Eds.) (2021). Body schema and body image: New directions. Oxford University Press, United Kingdom, p 85–98

[32] Guillot A, Collet C, Nguyen VA, Malouin F, Richards C, Doyon J (2009) Brain activity during visual versus kinesthetic imagery: an fMRI study. Hum Brain Mapp 30(7):2157–2172. 10.1002/hbm.20658 18819106 10.1002/hbm.20658 PMC6870928

[33] Chholak P, Niso G, Maksimenko VA, Kurkin SA, Frolov NS, Pitsik EN, Hramov EA, Pisarchik AN (2019) Visual and kinesthetic modes affect motor imagery classification in untrained subjects. Sci Rep 9(1):9838. 10.1038/s41598-019-46310-9 31285468 10.1038/s41598-019-46310-9 PMC6614413

[34] Kwon S, Kim J, Kim T (2023) Neuropsychological activations and networks while performing visual and kinesthetic motor imagery. Brain Sci 13(7):983. 10.3390/brainsci13070983 37508915 10.3390/brainsci13070983 PMC10377687

[35] Payne P, Levine PA, Crane-Godreau MA (2015) Somatic experiencing: using interoception and proprioception as core elements of trauma therapy. Front Psychol 6:93. 10.3389/fpsyg.2015.00093 25699005 10.3389/fpsyg.2015.00093 PMC4316402

[36] Keefe FJ, Rumble ME, Scipio CD, Giordano LA, Perri LM (2004) Psychological aspects of persistent pain: current state of the science. J Pain 5(4):195–211. 10.1016/j.jpain.2004.02.576 15162342 10.1016/j.jpain.2004.02.576

[37] Moseley GL (2005) Distorted body image in complex regional pain syndrome. Neurology 65(5):773–773. 10.1212/01.wnl.0000174515.07205.11 16157921 10.1212/01.wnl.0000174515.07205.11

[38] Moseley LG (2008) I can’t find it! Distorted body image and tactile dysfunction in patients with chronic back pain. Pain 140(1):239–243. 10.1016/j.pain.2008.08.001 18786763 10.1016/j.pain.2008.08.001

[39] Ozcan O, Karaali HK (2022) Kinesthetic and visual imagery in young adults with chronic neck pain. Sanamed. 10.5937/sanamed17-37885

[40] La Touche R, Grande-Alonso M, Cuenca-Martínez F, Gónzález-Ferrero L, Suso-Martí L, Paris-Alemany A (2019) Diminished kinesthetic and visual motor imagery ability in adults with chronic low back pain. PM&R 11(3):227–235. 10.1016/j.pmrj.2018.05.025 29908933 10.1016/j.pmrj.2018.05.025

[41] Lewis JS, Kersten P, McCabe CS, McPherson KM, Blake DR (2007) Body perception disturbance: a contribution to pain in complex regional pain syndrome (CRPS). PAIN® 133(1–3):111–119. 10.1016/j.pain.2007.03.013 17509761 10.1016/j.pain.2007.03.013

[42] Bowering KJ, Butler DS, Fulton IJ, Moseley GL (2014) Motor imagery in people with a history of back pain, current back pain, both, or neither. Clin J Pain 30(12):1070–1075. 10.1097/AJP.0000000000000066 24535054 10.1097/AJP.0000000000000066

[43] Bray H, Moseley GL (2011) Disrupted working body schema of the trunk in people with back pain. Br J Sports Med 45(3):168–173. 10.1136/bjsm.2009.061978 19887441 10.1136/bjsm.2009.061978

[44] Cole J, Paillard J (1995) Living without touch and peripheral information about body position and movement: Studies with deafferented subjects. In: Bermúdez JL, Marcel AJ, Eilan N (eds) The body and the self. The MIT Press, pp 245–266

[45] Kennett S, Eimer M, Spence C, Driver J (2001) Tactile-visual links in exogenous spatial attention under different postures: convergent evidence from psychophysics and ERPs. J Cogn Neurosci 13(4):462–478. 10.1162/08989290152001899 11388920 10.1162/08989290152001899

[46] Moseley GL, Wiech K (2009) The effect of tactile discrimination training is enhanced when patients watch the reflected image of their unaffected limb during training. PAIN® 144(3):314–319. 10.1016/j.pain.2009.04.030 19501965 10.1016/j.pain.2009.04.030

[47] Halicka M, Vittersø AD, Proulx MJ, Bultitude JH (2020) Neuropsychological changes in complex regional pain syndrome (CRPS). Behav Neurol 2020(1):4561831. 10.1155/2020/4561831 32399082 10.1155/2020/4561831 PMC7201816

[48] Muret D, Root V, Kieliba P, Clode D, Makin TR (2022) Beyond body maps: Information content of specific body parts is distributed across the somatosensory homunculus. Cell reports. 10.1016/j.celrep.2022.110523 35294887 10.1016/j.celrep.2022.110523 PMC8938902

[49] Watson JC, Sandroni P (2016) Central neuropathic pain syndromes. Mayo clinic proceedings. Elsevier, pp 372–385 10.1016/j.mayocp.2016.01.017 26944242

[50] Díaz-Mohedo E, González-Roldán G, Muñoz-Gámez I, Padilla-Romero V, Castro-Martín E, Cabrera-Martos I, Sánchez-García C (2023) Implicit motor imagery for chronic pelvic pain: A cross-sectional case–control study. J Clin Med 12(14):4738. 10.3390/jcm12144738 37510853 10.3390/jcm12144738 PMC10380828

[51] Tsakiris M, Longo MR, Haggard P (2010) Having a body versus moving your body: neural signatures of agency and body-ownership. Neuropsychologia 48(9):2740–2749. 10.1016/j.neuropsychologia.2010.05.021 20510255 10.1016/j.neuropsychologia.2010.05.021

[52] Gong G, Rosa-Neto P, Carbonell F, Chen ZJ, He Y, Evans AC (2009) Age-and gender-related differences in the cortical anatomical network. J Neurosci 29(50):15684–15693. 10.1523/JNEUROSCI.2308-09.2009 20016083 10.1523/JNEUROSCI.2308-09.2009 PMC2831804

